# Changes in Circulating Inflammatory and Cardiac Biomarkers at Three-Month Follow-Up After CABG

**DOI:** 10.3390/jcm15208150

**Published:** 2026-10-20

**Authors:** Ruben Flavius Mateaş, Ştefan Cristian Vesa, Raluca Pop, Antonia Eugenia Macarie, Victor Furculiță, Carolina Borș, Alexandra Dădârlat Pop, Simona Manole, Dan Ion Bindea, Florin Dumitru Bora, Adrian Ștef, Daciana Elena Popa, Adrian Molnar

**Affiliations:** 1 Department of Cardiovascular Surgery, “Niculae Stăncioiu” Heart Institute, 19–21 Moților Street, 400001 Cluj-Napoca, Romania; 2 Division of Cardiology, Faculty of Medicine, “Iuliu Hațieganu” University of Medicine and Pharmacy, 8 Victor Babeș Street, 400012 Cluj-Napoca, Romania; 3 Division of Pharmacology, Toxicology and Clinical Pharmacology, Department of Morpho-Functional Sciences, “Iuliu Hațieganu” University of Medicine and Pharmacy, 8 Victor Babeș Street, 400347 Cluj-Napoca, Romania; 4 Division of Geriatrics and Gerontology, Department of Medical Specialties, “Iuliu Hațieganu” University of Medicine and Pharmacy, 8 Victor Babeș Street, 400347 Cluj-Napoca, Romania; 5 Department of Cardiology, “Niculae Stăncioiu” Heart Institute, 19–21 Moților Street, 400002 Cluj-Napoca, Romania; 6 Department of Radiology and Medical Imaging, “Niculae Stăncioiu” Heart Institute, 400001 Cluj-Napoca, Romania; 7 Department of Radiology and Medical Imaging, “Iuliu Hațieganu” University of Medicine and Pharmacy, 8 Victor Babeș Street, 400012 Cluj-Napoca, Romania; 8 Laboratory of Chromatography, Advanced Horticultural Research Institute of Transylvania, Faculty of Horticulture and Business in Rural Development, University of Agricultural Sciences and Veterinary Medicine of Cluj-Napoca, 400372 Cluj-Napoca, Romania; 9 Clinical Department of Anesthesia and Intensive Care, “Niculae Stăncioiu” Heart Institute, 19–21 Moților Street, 400001 Cluj-Napoca, Romania; 10 Discipline of Anesthesia and Intensive Care II, “Iuliu Hațieganu” University of Medicine and Pharmacy, 8 Victor Babeș Street, 400012 Cluj-Napoca, Romania; 11 Department of Cardiology, HELORA University Hospital—Jolimont Site, 7100 La Louvière, Belgium

**Keywords:** CABG, inflammatory biomarkers, cardiac biomarkers

## Abstract

**Background**. Biomarkers associated with inflammation and the cardiovascular system are altered during the perioperative and immediate postoperative periods after coronary artery bypass grafting (CABG) but less is known about the changes occurring during the intermediate recovery phase. We measured levels of 7 inflammatory and cardiovascular biomarkers in CABG patients between the preoperative phase and follow-up three months after hospital discharge. We hypothesized that the circulating inflammatory and cardiac biomarker profile at three-month follow-up would differ from the preoperative profile. **Methods**. A prospective longitudinal observational cohort included 45 adults who underwent elective coronary artery bypass grafting between May 2025 and February 2026; 40 subjects had analyzable paired preoperative and three-month post-discharge biomarker samples. Plasma levels of the inflammatory and cardiovascular biomarkers, including high-sensitivity C-reactive protein (hsCRP), tumor necrosis factor alpha (TNF-α), interleukin-6 (IL-6), interleukin-18 (IL-18), resistin, N-terminal pro-B-type natriuretic peptide (NT-proBNP) and cardiac troponin I (cTnI) were measured on the day before surgery and at three-month follow-up after hospital discharge. **Results**. The analyzed cohort had a median age of 69.0 years and included 33 men (82.5%). Following correction for multiple testing, only IL-18 (from 119.6 [87.9–155.0] to 163.6 [111.9–228.0], *p* = 0.007 [adjusted *p* = 0.025]) and NT-proBNP (221.0 [135.3–424.8] to 372.6 [213.4–632.0], *p* = 0.001 [adjusted *p* = 0.007]) had statistically significant longitudinal increases. Secondary mixed-effects model analyses produced directionally consistent results, with follow-up-to-baseline ratios of 1.26 [95% confidence interval (CI) 1.05–1.52; *p* = 0.015] for IL-18 and 1.48 [95% CI 1.15–1.90; *p* = 0.003] for NT-proBNP. The levels of hsCRP, TNF-α, IL-6, resistin and cTnI did not change significantly over time. **Conclusions**. At three months after discharge, significant increases were confined to IL-18 and NT-proBNP rather than occurring across the broader circulating biomarker panel. These findings extend the limited prospective evidence on intermediate post-CABG biomarker changes and support further evaluation of IL-18 and NT-proBNP as candidate markers of postoperative recovery.

## 1. Introduction

Coronary artery bypass grafting (CABG) is a well-established myocardial revascularization technique in coronary artery disease, particularly in selected patients with left main disease, multivessel disease, diabetes, or left ventricular dysfunction [1]. In addition to revascularization, myocardial ischemia–reperfusion injury, surgical trauma, contact of blood with the extracorporeal circuit, the activation of leukocytes and the complement cascade, endothelial damage, and the resulting oxidative stress evoke systemic biological responses. Even though inflammatory activation may be more intense during on-pump CABG because of extracorporeal circulation, such inflammatory activation is also documented after off-pump CABG and thus is not exclusively related to cardiopulmonary bypass [2,3,4].

The post-CABG course is characterized by a significant yet heterogeneous early-onset release of cytokines and acute-phase proteins. Tumor necrosis factor alpha (TNF-α) responses are characterized by an early and transient increase while interleukin-6 (IL-6) and C-reactive protein elevations tend to last longer before returning toward baseline [2,5]. Nevertheless, during postoperative recovery, the course is not characterized solely by changes in circulating inflammatory parameters but also by cellular and extracellular matrix changes that may continue for several weeks after discharge [6,7].

Interleukin-18 (IL-18), a member of the interleukin-1 (IL-1) cytokine family, is pro-inflammatory, and its activity is controlled in part by IL-18 binding protein (IL-18BP) [8]. Immediate postoperative investigations into the behavior of circulating IL-18 in CABG patients have revealed dynamic postoperative changes in IL-18, and experimental studies have implicated IL-18 in myocardial dysfunction and remodeling [9,10]. There have been only limited reports investigating IL-18 in the later recovery period following CABG [9].

Post-CABG cardiovascular recovery remains complex. N-terminal pro-B-type natriuretic peptide (NT-proBNP), a marker circulating in the blood associated with myocardial wall stress, tends to rise in the immediate postoperative period and has been linked with impaired cardiac function and greater intensive care requirements following surgery [11,12]. Nevertheless, the importance of this biomarker in later recovery is not as clear, as its level can be affected by persistent or evolving changes in cardiac function, ventricular loading conditions, remodeling, and renal function [13,14,15,16]. Outside the setting of acute illness, high-sensitivity cardiac troponin T and NT-proBNP have been linked to certain measures of subclinical cardiovascular dysfunction, including arterial stiffness and cardiac structural or functional abnormalities [17]. While these findings cannot be directly applied to patients recovering from CABG, they provide a rationale to further explore whether changes in cardiac biomarkers during the intermediate recovery phase may identify subclinical abnormalities or be associated with subsequent clinical outcomes.

While the biological effects of CABG surgery may not occur solely around the immediate postoperative period, most of the research in this area has focused on circulating biomarkers in the short-term recovery phase; even fewer studies have included simultaneous assessment of cardiac and inflammatory markers. Assessment of these biomarkers three months after discharge may help ascertain whether a broad normalization of the circulating biomarker profile occurs or whether there are selective changes in specific inflammatory and/or cardiovascular processes. Accordingly, the primary aim of the current study was to investigate longitudinal changes in circulating markers of inflammation (high-sensitivity C-reactive protein [hsCRP], TNF-α, IL-6, IL-18, and resistin) and cardiac biomarkers (NT-proBNP and cardiac troponin I) from the preoperative assessment to the three-month post-discharge follow-up.

## 2. Materials and Methods

### 2.1. Study Design and Population

This prospective longitudinal observational cohort study was performed at the Heart Institute, Cluj-Napoca, Romania. A total of 52 patients scheduled for elective coronary revascularization were assessed for eligibility between May 2025 and February 2026. Four patients declined study participation, 2 subsequently declined CABG and were discharged, and 1 underwent percutaneous coronary intervention instead of CABG. Thus, 45 patients undergoing CABG were included at baseline. The Ethics Committee of “Iuliu Hațieganu” University of Medicine and Pharmacy, Cluj-Napoca, Romania, approved the study [No. 59/01.04.2024] before the beginning of the project. Written informed consent was obtained from all patients before inclusion in the study.

The inclusion criteria were:

- Age ≥ 18 years;
- Ischemic heart disease with a clear indication for elective isolated CABG;
- Provision of written informed consent.

The exclusion criteria were:

- Emergency revascularization;
- Concomitant valve surgery or other cardiac surgical procedures;
- Unwillingness to participate in the study.

### 2.2. Clinical and Perioperative Data Collection

On study enrollment, demographic data, including age and sex; clinical information, including body mass index (BMI); medical history regarding cardiovascular disease and comorbidities; and current prescribed medications (renin–angiotensin–aldosterone system (RAAS) inhibitors, beta-blockers, calcium channel blockers, angiotensin receptor–neprilysin inhibitors (ARNIs), diuretics, statins, anticoagulants, antiplatelet agents, amiodarone, and digoxin) were collected. Recorded comorbidities included chronic kidney disease, preoperative atrial fibrillation (AF), prior myocardial infarction (MI), and prior stroke.

Perioperatively obtained data included the number of bypass grafts performed, operative technique (on-pump vs. off-pump CABG), and cardiopulmonary bypass duration in on-pump patients. All on-pump procedures were performed using conventional cardiopulmonary bypass; no minimally invasive extracorporeal circulation (MiECC) system was used. In on-pump patients, aortic cross-clamp time and the type and volume of cardioplegia were also recorded. Del Nido cardioplegia was used in all 27 on-pump procedures, with a median total administered volume of 1000 mL (IQR 1000–1000 mL; range 1000–2400 mL), including any additional doses administered during surgery. Postoperative data included intensive care unit (ICU) stay, total hospital stay, postoperative AF, postoperative stroke, postoperative MI, major postoperative bleeding requiring surgical reintervention, in-hospital mortality, prescribed drugs at hospital discharge, and subsequent therapy. Postoperative AF was defined as new-onset atrial fibrillation occurring after CABG during the index hospitalization and documented by electrocardiography or continuous cardiac monitoring.

### 2.3. Echocardiographic Assessment

Baseline transthoracic echocardiographic parameters recorded included left ventricular end-systolic volume (LVESV), left ventricular end-diastolic volume (LVEDV), left ventricular ejection fraction (LVEF), left atrial volume (LAV), and tricuspid annular plane systolic excursion (TAPSE). Echocardiographic examinations were performed using a GE Vivid E95 (v206) ultrasound system by two experienced examiners.

### 2.4. Laboratory Analyses

Blood samples at V1 and V2 were collected under comparable conditions, in the morning after an overnight fast. Baseline blood samples (V1) were collected on the day before CABG, and follow-up blood samples (V2) were collected three months after hospital discharge. Blood samples were collected in ethylenediaminetetraacetic acid (EDTA) tubes, and plasma was separated by centrifugation at 1000× *g* for 10 min within 30 min of sample collection. Following separation, the plasma was aliquoted and stored at −80 °C until analysis. The characteristics of the enzyme-linked immunosorbent assay (ELISA) kits used for biomarker measurement, including manufacturer, catalog number, detection range, and analytical sensitivity, are summarized in Table 1. All analyses were performed according to the manufacturers’ protocols. Paired preoperative (V1) and follow-up (V2) samples from each patient were analyzed within the same analytical batch for each biomarker to minimize inter-batch variability. Optical density was measured using an 800 TS microplate reader (Agilent Technologies Inc., Santa Clara, CA, USA), while an automated BioTek 50 TS microplate washer (Agilent Technologies Inc., Santa Clara, CA, USA) was used for the plate washing steps. ELISA concentrations were calculated automatically using the instrument software by fitting the standard curves with a four-parameter logistic (4PL) regression model. Samples were analyzed undiluted unless otherwise specified. For hsCRP, plasma samples were diluted 1:10,000 before analysis. The concentrations interpolated from the 4PL calibration curve were multiplied by 10,000 to obtain the concentrations in the original plasma samples, which were subsequently converted from pg/mL to mg/L for reporting.

### 2.5. Statistical Analysis

Data were analyzed using R 4.6.0 software. The distribution of continuous variables was assessed using the Shapiro–Wilk test and by visual inspection of their distributions. Because several continuous variables, especially biomarker concentrations, showed non-normal distributions and given the sample size, continuous variables are presented as median and interquartile range (Q1–Q3). Categorical variables are presented as absolute frequencies and percentages.

Longitudinal differences in the seven circulating biomarkers between the preoperative measurement obtained on the day before CABG (V1) and the follow-up measurement obtained three months after hospital discharge (V2) were determined using the paired Wilcoxon signed-rank test. The rank-biserial correlation was calculated as an effect-size measure for each paired Wilcoxon signed-rank comparison; no between-variable correlation analysis was performed. The *p* values were adjusted for multiple comparisons using the Benjamini–Hochberg false discovery rate (FDR) procedure. For the primary comparisons of the seven biomarkers, statistical significance was determined on the basis of the FDR-adjusted *p* values, with an adjusted *p* value < 0.05 considered statistically significant.

Linear mixed-effects models were constructed separately for each circulating biomarker. The biomarkers were logarithmically transformed prior to modelling due to the right-skewed nature of their distributions. Model coefficients were exponentiated to represent the V2-to-V1 ratio, with 95% confidence intervals (CIs), providing an estimate of the proportional change in biomarker concentration over follow-up. These models were considered secondary supportive analyses, and their *p* values were reported without adjustment for multiple comparisons. Statistical inference for the primary longitudinal biomarker comparisons was based on the FDR-adjusted paired Wilcoxon tests.

To investigate whether longitudinal biomarker changes differed according to selected patient characteristics, exploratory effect-modification analyses using linear mixed-effects models were subsequently performed for IL-18 and NT-proBNP. Candidate modifiers represented relevant demographic, clinical, echocardiographic, operative, or pharmacological variables of interest, including age, sex, body mass index, creatinine, preoperative atrial fibrillation, prior myocardial infarction, surgical strategy, left ventricular ejection fraction, and selected medications prescribed at hospital discharge. Candidate effect modifiers were selected a priori on the basis of clinical plausibility and their potential relevance to inflammatory or cardiac biomarker changes, rather than through data-driven variable selection. Age and sex were prespecified as adjustment covariates because of their potential influence on biomarker concentrations and postoperative recovery. For each potential modifying variable, a separate linear mixed-effects model was built. Log-transformed biomarker concentration was modelled as a function of time, the candidate modifier, and the time × modifier interaction, together with a patient-specific random intercept. Models were adjusted for age and sex, unless age or sex was the modifier itself. Continuous modifier variables were standardized before analysis, and as such, interaction estimates for continuous predictors represented the change in the V2-to-V1 ratio associated with a one-standard-deviation increase in each continuous modifier. For binary modifier variables, the estimates represented the ratio between the V2-to-V1 changes in the two categories. Interaction *p* values were adjusted separately for each biomarker using the Benjamini–Hochberg FDR procedure. The effect-modification analyses were exploratory, and the findings were interpreted based on the magnitude of the effects, including 95% CIs and multiplicity-adjusted *p* values. These analyses were interpreted cautiously in view of the modest sample size and limited power to detect interaction effects; therefore, non-significant interactions were not considered evidence of absence of effect modification.

We considered a *p* value < 0.05 to represent statistical significance for analyses where no multiplicity correction was required and used FDR-adjusted *p* values for inference in multiple-testing settings.

## 3. Results

**Figure 1:**
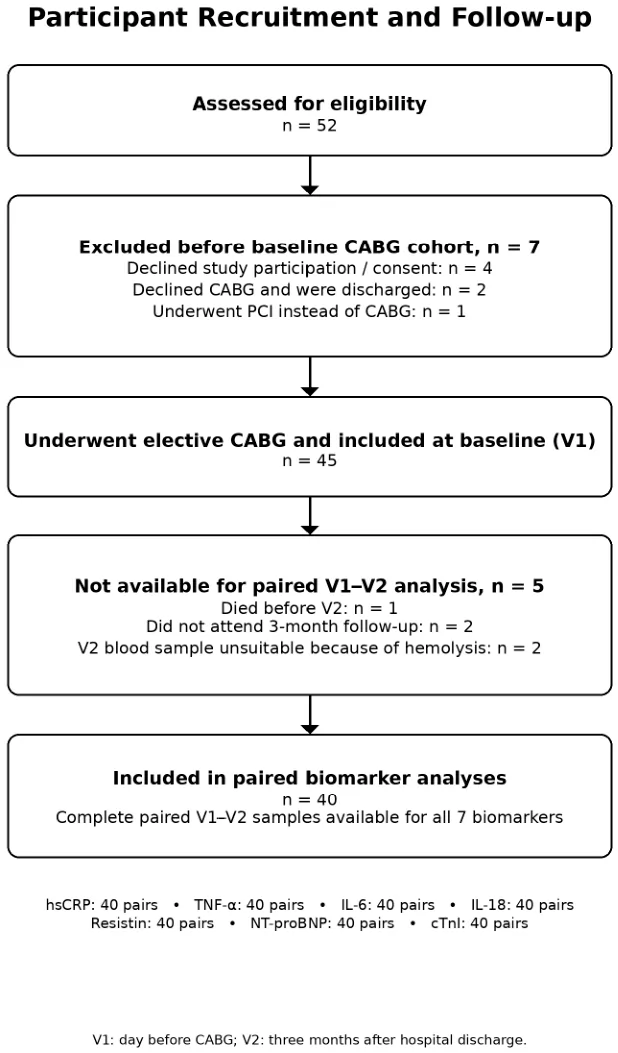
Flow diagram of participant recruitment and follow-up. CABG, coronary artery bypass grafting; PCI, percutaneous coronary intervention; V1, baseline blood sampling on the day before CABG; V2, follow-up blood sampling three months after hospital discharge.

Of the 45 patients included at baseline, one died before the follow-up assessment due to cerebral hemorrhage, two did not attend the three-month follow-up, and two had follow-up blood samples unsuitable for analysis because of hemolysis. Consequently, 40 patients had complete analyzable paired V1 and V2 samples and were included in the longitudinal biomarker analyses (Figure 1). Their perioperative demographic, clinical, and echocardiographic characteristics are reported in Table 2. The median age of the patients was 69.0 years (61.0–71.5). The cohort included 33 men (82.5%) and 7 women (17.5%); hypertension was present in 35 patients (87.5%) and previous myocardial infarction in 14 (35.0%). Median LVEF was 54.0%, and 27 procedures (67.5%) were performed on-pump. Postoperative AF occurred in 30.0% of patients, but no intrahospital deaths, MIs or strokes were reported.

Table 3 outlines the treatments prescribed to our patients at hospital discharge. Most patients received a beta-blocker; however, RAAS inhibitors, statins, and antiplatelet agents were also prescribed to most patients. No changes in cardiovascular medication regimens were documented between hospital discharge and the three-month follow-up.

Table 4 presents longitudinal changes in the biomarker panel. Following correction for multiple testing using the Benjamini–Hochberg false discovery rate procedure, NT-proBNP and IL-18 were the only markers that showed significant increases between V1 and V2 in this analysis, whereas all other markers showed nonsignificant changes. Linear mixed-effects models fitted to log-transformed concentrations produced results similar to those displayed in Table 4, showing nominally significant increases only for NT-proBNP and IL-18 in the V1-to-V2 comparison (V2/V1 ratio 1.48, 95% CI 1.15–1.90; *p* = 0.003; V2/V1 ratio 1.26, 95% CI 1.05–1.52; *p* = 0.015, respectively).

Effect modification by demographic (age, sex, BMI), clinical, echocardiographic, operative, and pharmacological variables (creatinine, preoperative AF, prior MI, surgical strategy, LVEF, and selected medication categories) was investigated to characterize whether certain factors modified the pattern of IL-18 over time. No tested interaction remained statistically significant after adjustment for multiple comparisons (Table 5).

The exploratory analyses of the V1-to-V2 change in NT-proBNP yielded similar findings. No examined variable significantly modified the change in NT-proBNP after adjustment for multiple comparisons (Table 6).

## 4. Discussion

The two main findings of this study were the significant increases in circulating IL-18 and NT-proBNP at the three-month post-discharge follow-up (V2), while hsCRP, IL-6, TNF-α, resistin, and cardiac troponin I showed no significant changes after correction for multiple comparisons. These findings were supported by the primary paired comparisons, while secondary linear mixed-effects models yielded directionally consistent V2-to-V1 ratios of 1.26 for IL-18 and 1.48 for NT-proBNP. Overall, the post-CABG circulating inflammatory and cardiac biomarker profile was characterized by selective rather than global changes in biomarker levels. The absence of significant change in IL-6, TNF-α, and hsCRP at three months following CABG is consistent with typical postsurgical time courses.

These inflammatory markers may show immediate elevations (hours to days) after surgery, tending to return toward baseline levels thereafter. However, absence of sustained increases in the circulation does not signify resolution of the immune response as monocyte phenotype and ex vivo cytokine stimulation remain altered several weeks after bypass surgery, and the latter is the case even when circulating IL-6 is no longer significantly elevated. For this reason, the absence of significant changes in these cytokines should be interpreted not as complete recovery but as the absence of a significant difference in their circulating levels at follow-up relative to baseline [2,5,6,18]. Therefore, the absence of significant V1-to-V2 changes in hsCRP, IL-6, and TNF-α in our cohort is consistent with the prior literature describing these markers as predominantly early postoperative responders, with subsequent decreases toward baseline during convalescence [2,5,18]. Due to the lack of early postoperative time points in our study, we are unable to evaluate potential transient elevations [2,5].

Resistin also did not show a significant change between the preoperative and three-month assessments. Other cardiac surgery reports describe resistin mostly as an acute perioperative mediator, with increases reported during the immediate postoperative period together with oxidative stress, adipose-tissue inflammatory activity, and myocardial damage. The absence of a significant difference in resistin at follow-up in our cohort seems compatible with this concept, as it is mainly a response to acute metabolic and ischemia–reperfusion injury, rather than a marker of sustained late systemic inflammation [19,20]. Accordingly, our finding extends the predominantly perioperative observations reported previously by showing no significant difference between preoperative and three-month concentrations, although the intervening time course remains unknown.

However, the pattern of IL-18 behavior may be distinct. Prior studies on CABG have revealed marked increases in concentrations in the immediate postoperative period, following both on-pump and off-pump surgery, with higher levels reported after cardiopulmonary bypass (CPB) and associations with markers of systemic and endothelial injury [21]. No statistically significant interaction between surgical strategy and time was detected for IL-18 after correction for multiple comparisons, although the limited sample size does not exclude clinically relevant differences. These observations suggest that the increase in IL-18 observed at follow-up was not clearly attributable to a single measured covariate or to CPB exposure alone. The small cohort size and small subgroup sizes limit the ability to detect smaller interaction effects.

It is crucial that the early and late IL-18 responses should not be assumed to represent the same continuous elevation. There is some evidence to support a dramatic peak hours after CABG, with free IL-18 levels then dropping below the preoperative value during the first postoperative day, due to concurrent changes in total IL-18 concentrations and circulating IL-18 binding protein. As only preoperative and three-month measurements were available in our study, the intervening behavior of IL-18 levels cannot be accurately determined, and it may have returned toward baseline before rising again or even have followed a more complicated trajectory. The observed value should therefore be viewed as an increase at three months relative to the preoperative value and not as a persistence of the acute postoperative IL-18 response [9].

Previous experimental and clinical evidence has associated IL-18 with inflammasome-related signaling and myocardial remodeling [7,22,23,24]. However, in our current study we only measured circulating total IL-18 and did not assess inflammasome activation, free IL-18, myocardial fibrosis, or serial cardiac structural or functional changes. The increase in IL-18 we observed should therefore be interpreted solely as a change in a circulating biomarker and not as evidence of a specific remodeling mechanism.

Regarding NT-proBNP, the median concentration increased from 221.0 to 372.6 pg/mL, with statistical significance maintained after adjustment for multiple comparisons. The V2/V1 ratio determined using the linear mixed-effects model was also concordant at 1.48 (approximately a 48% higher geometric mean concentration at V2 than at V1). Several studies have evaluated cardiac remodeling with serial echocardiographic assessments, but only a few have focused on post-CABG outcomes. Most previous clinical studies of NT-proBNP after CABG investigated the early postoperative period, during which NT-proBNP rises rapidly in association with surgical stress, ischemia–reperfusion injury, and early postoperative cardiac dysfunction.

In our cohort, the level of NT-proBNP at the three-month post-CABG stage should be viewed as a temporally distinct finding from the early postoperative rise in NT-proBNP after cardiac surgery rather than necessarily the same ongoing elevation. Marked increases in postoperative NT-proBNP levels after CABG have been reported by several research groups and have been associated with myocardial damage, postoperative heart failure, prolonged ICU stay, and additional circulatory support in early postoperative days [11,12,25].

Since NT-proBNP is short-lived in circulation, the higher V2 concentration cannot simply reflect residual peptide released in the immediate postoperative period, but this does not clarify the mechanism responsible for the higher concentration. In prior CABG studies, factors influencing NT-proBNP in later recovery include ventricular loading and remodeling, preexisting cardiac phenotype, and renal function [13,14,15,16,26,27]. There was no routine assessment of follow-up echocardiography, cardiovascular hemodynamics, or renal function in this study, and we therefore cannot definitively attribute the higher V2 concentration to abnormal hemodynamics or incomplete cardiovascular recovery.

No time × modifier interactions remained statistically significant after FDR correction. However, the limited sample size and statistical power do not allow us to exclude potentially clinically relevant effect modification. It is improbable that ongoing myocardial necrosis would explain the increased NT-proBNP level at the three-month post-discharge follow-up relative to baseline.

There was no significant change in cardiac troponin I between V1 and V2, and no postoperative MI occurred during hospitalization. Cardiac troponin I release following CABG is predominantly an early postoperative event, with peak concentrations occurring during the first postoperative hours and subsequent decline in uncomplicated cases [28]. Hence, the absence of a significant V1-to-V2 difference at three months in our cohort appears consistent with resolution of the acute perioperative myocardial injury signal, although it does not exclude residual ischemia or other forms of myocardial stress. A stable troponin I level may not provide sufficient information to exclude ongoing ischemia or microvascular dysfunction, altered ventricular filling pressures, or altered ventricular wall stress, but it makes ongoing myocardial necrosis a less likely explanation for the increase in NT-proBNP. Altered myocardial wall stress or hemodynamic conditions may represent alternative explanations, although these were not directly assessed. Among the 40 patients included in the paired analysis, no in-hospital deaths or postoperative strokes were recorded, and reoperation for major postoperative hemorrhage was required in only one patient. Postoperative AF occurred in 30.0% of patients in the study group, a prevalence consistent with that reported in contemporary CABG studies [29]. The biomarker changes at follow-up therefore occurred in the context of an overall largely favorable early postoperative course and are unlikely to be explained solely by major perioperative ischemic complications. However, it was outside the scope of this study to clarify the influence of specific postoperative complications on individual biomarker changes.

Five of the seven biomarkers showed no significant V1-to-V2 change, whereas IL-18 and NT-proBNP increased significantly; secondary mixed-effects models yielded directionally consistent estimates. The IL-18 increase may be compatible with inflammatory processes, whereas the NT-proBNP increase may reflect factors influencing natriuretic peptide production during postoperative recovery; however, the present study cannot establish the mechanisms underlying either change. These increases in NT-proBNP and IL-18 levels should not be construed as evidence of a shared mechanism. Their mutual relationship was not tested in the present study; therefore, a possible correlation would not inherently confirm a common causal pathway.

From a clinical perspective, these findings do not justify the routine use of IL-18 and NT-proBNP for post-CABG surveillance but do suggest that these potential markers should be further evaluated during intermediate postoperative recovery and in relation to longer-term outcomes. While NT-proBNP can be widely measured clinically, interpretation may be affected by age, renal impairment, atrial fibrillation, volume status, and baseline cardiac status. Although potentially more specific as an inflammatory marker, IL-18 is not widely available in the clinical setting; there are no defined thresholds for clinical decisions, and free biologically active IL-18 may be more relevant to measure than total IL-18, as measured in the present study. In comparison, inflammatory indices derived from routine complete blood count (CBC) parameters, such as the neutrophil-to-lymphocyte ratio, platelet-to-lymphocyte ratio, and systemic immune-inflammation index, are inexpensive and readily available markers that have been associated with postoperative outcomes after CABG. Although inexpensive and easily measured, these indices are nonspecific and may reflect a wide range of inflammatory or systemic responses. It remains to be determined whether IL-18 or NT-proBNP provides clinically meaningful incremental information beyond these routinely available markers [30,31]. Further studies are needed to determine whether these markers provide clinically meaningful information regarding outcomes such as incident heart failure, adverse ventricular remodeling, recurrent ischemia, atrial fibrillation, readmission, and mortality.

It is appropriate to note certain limitations of our study. First, it was a single-center observational study with only a modestly sized sample, which limits the generalizability of the findings and the statistical power of subgroup or interaction analyses. Second, without a contemporaneous non-operated control group, we cannot confidently attribute the changes observed in the biomarkers to CABG because it is possible that temporal biological variation, clinical progression or recovery of the index cardiac and/or concomitant conditions, intercurrent events, and changes in medications may all have influenced changes in the measured biomarkers. Third, biomarkers were only assessed one day preoperatively and at the three-month post-discharge follow-up. The study thus describes the changes in measures obtained one day pre-CABG compared with the three-month post-discharge follow-up but does not characterize the intervening time course, timing of onset, or persistence of the observed changes. Fourth, while follow-up echocardiography could have provided information on systolic and diastolic left ventricular (LV) function, myocardial strain and deformation, right ventricular systolic function, and LV filling pressures, it was not performed; residual myocardial ischemia and graft patency were also not assessed. Hence, any interpretation regarding the mechanisms underlying the increase in NT-proBNP is speculative. No follow-up creatinine measurements or other renal function data were available at the three-month assessment. Therefore, changes in renal function between V1 and V2 could not be evaluated, and alterations in renal clearance cannot be excluded as a potential contributor to the observed increase in NT-proBNP. Fifth, circulating total IL-18 was assessed, but IL-18BP and free biologically active IL-18 were not measured, and downstream signaling or inflammasome activation was also not directly examined; thus, there is no direct evidence that biologically active IL-18 increased or that the NLR family pyrin domain containing 3 (NLRP3) inflammasome was activated.

In addition, the presence of both on- and off-pump CABG patients could have contributed to the heterogeneity in the inflammatory response. While surgical strategy was analyzed as a possible effect modifier and no statistically significant interaction was detected after adjustment for multiple comparisons, we had limited statistical power to exclude clinically important differences in this subgroup analysis.

## 5. Conclusions

At the three-month follow-up, IL-18 and NT-proBNP were the only biomarkers showing statistically significant increases. These findings do not justify the routine use of either biomarker for monitoring patients after CABG but do identify them as candidate markers for further evaluation during intermediate postoperative recovery. Larger prospective studies should establish whether these markers provide incremental clinical information and are associated with later heart failure or adverse ventricular remodeling, recurrent ischemia, atrial fibrillation, readmission, or mortality.

## Figures and Tables

**Table 1:** Characteristics of ELISA kits used for biomarker measurements.

Biomarker	Manufacturer	Catalog Number	Detection Range	Sensitivity	Intra-Assay CV (%)	Inter-Assay CV (%)
NT-proBNP	ELK Biotechnology, Wuhan, China	ELK1212	39.07–2500 pg/mL	15 pg/mL	<8%	<10%
hsCRP	Elabscience Biotechnology Inc., Houston, TX, USA	E-EL-H5134	15.63–1000 pg/mL	9.38 pg/mL	4.47–4.64%	6.44–8.95%
IL-18	Elabscience Biotechnology Inc.	E-EL-H0253	15.63–1000 pg/mL	9.38 pg/mL	5.06–6.03%	6.45–7.43%
Resistin	Elabscience Biotechnology Inc.	E-EL-H1213	31.25–2000 pg/mL	18.75 pg/mL	4.29–5.11%	6.49–8.16%
cTnI	Invitrogen/Thermo Fisher Scientific (Walthened, CO, USA)	EHTNNI3	0.38–625 pg/mL	0.38 pg/mL	<10%	<12%
IL-6	Invitrogen/Thermo Fisher Scientific	BMS213-2	1.56–100 pg/mL	0.92 pg/mL	3.4%	5.2%
TNF-α	Invitrogen/Thermo Fisher Scientific	88-7346-88	4–500 pg/mL	4 pg/mL	<6%	<10%

Abbreviations: cTnI, cardiac troponin I; hsCRP, high-sensitivity C-reactive protein; IL, interleukin; NT-proBNP, N-terminal pro-B-type natriuretic peptide; TNF-α, tumor necrosis factor alpha. Intra-assay and inter-assay CVs are manufacturer-reported precision data.

**Table 2:** Demographic, clinical, echocardiographic, and perioperative characteristics of the CABG cohort.

Characteristic		CABG Cohort (n = 40)
Age, years		69.0 (61.0, 71.5)
Sex	Female	7 (17.5%)
	Male	33 (82.5%)
Body mass index (BMI), kg/m^2^		30.7 (25.6, 34.2)
Hypertension		35 (87.5%)
Preoperative AF		8 (20.0%)
Previous MI		14 (35.0%)
Previous stroke		2 (5.0%)
Chronic kidney disease		1 (2.5%)
Serum urea (mg/dL)		38.9 (32.5, 54.8)
Serum creatinine, mg/dL		0.9 (0.8, 1.0)
LVEF, %		54.0 (45.0, 59.0)
LV end-diastolic volume, mL		130.5 (107.0, 158.5)
LV end-systolic volume, mL		58.0 (44.0, 77.5)
Left atrial volume, mL		65.0 (53.0, 80.0)
TAPSE, mm		20.0 (20.0, 21.0)
Number of bypass grafts		2.0 (2.0, 3.0)
Surgical strategy	On-pump	27 (67.5%)
	Off-pump	13 (32.5%)
Cardiopulmonary bypass time, min		104.0 (78.0, 140.0)
Aortic cross-clamp time, min		62.0 (49.0, 98.0)
Cardioplegia—del Nido		27 (100%)
Total cardioplegia volume, mL		1000 (1000, 1000)
ICU stay, days		3.0 (2.0, 3.5)
Total hospital stay, days		12.0 (10.5, 15.0)
Postoperative atrial fibrillation		12 (30.0%)
Major bleeding requiring reintervention		1 (2.5%)
Postoperative stroke		0 (0.0%)
Postoperative myocardial infarction		0 (0.0%)
Death		0 (0.0%)

Cardiopulmonary bypass time and cardioplegia data are reported for the 27 patients undergoing on-pump CABG. Aortic cross-clamp time was available for 25 of these patients. Abbreviations: AF, atrial fibrillation; BMI, body mass index; CABG, coronary artery bypass grafting; ICU, intensive care unit; LV, left ventricular; LVEF, left ventricular ejection fraction; MI, myocardial infarction; TAPSE, tricuspid annular plane systolic excursion.

**Table 3:** Pharmacological treatment prescribed at hospital discharge.

Medication Class	Prescribed at Discharge, n (%)
RAAS inhibitor	32 (80%)
Beta-blocker	39 (97.5%)
Calcium-channel blocker	9 (22.5%)
Loop diuretic	10 (25.0%)
Mineralocorticoid receptor antagonist (MRA)	8 (20.0%)
Statin	40 (100%)
Aspirin	35 (87.5%)
P2Y12 inhibitor	29 (72.5%)
Dual antiplatelet therapy	29 (72.5%)
Oral anticoagulant	20 (50%)
Amiodarone	14 (35.0%)
Digoxin	1 (2.5%)

Abbreviations: MRA, mineralocorticoid receptor antagonist; RAAS, renin–angiotensin–aldosterone system.

**Table 4:** Longitudinal changes in circulating biomarkers.

Biomarker	V1, Median (Q1, Q3)	V2, Median (Q1, Q3)	*p*	FDR-Adjusted *p*	Rank-Biserial Effect Size
NT-proBNP (pg/mL)	221.0 (135.3, 424.8)	372.6 (213.4, 632.0)	0.001	0.007	0.590
IL-18 (pg/mL)	119.6 (87.9, 155.0)	163.6 (111.9, 228.0)	0.007	0.025	0.493
hsCRP (mg/L)	5.1 (4.1, 6.4)	4.9 (4.1, 6.2)	0.405	0.687	0.151
Troponin I (pg/mL)	32.8 (16.6, 48.6)	30.2 (16.6, 53.4)	0.519	0.687	0.117
IL-6 (pg/mL)	14.0 (4.3, 32.9)	10.8 (5.9, 24.5)	0.582	0.687	0.100
TNF-alpha (pg/mL)	13.9 (7.5, 32.8)	14.6 (5.4, 27.2)	0.628	0.687	−0.088
Resistin (pg/mL)	128.9 (89.4, 183.8)	139.1 (90.2, 191.7)	0.687	0.687	−0.073

The *p* values were adjusted across the seven biomarker comparisons using the Benjamini–Hochberg false discovery rate procedure. Statistical significance was determined using the FDR-adjusted *p* values. Abbreviations: FDR, false discovery rate; hsCRP, high-sensitivity C-reactive protein; IL, interleukin; NT-proBNP, N-terminal pro-B-type natriuretic peptide; TNF-α, tumor necrosis factor alpha; V1, preoperative measurement; V2, three-month post-discharge follow-up measurement.

**Table 5:** Effect modifiers of longitudinal IL-18 changes.

Predictor	Group or Scale	N	Adjusted V2/V1 Ratio (95% CI)	Interaction Ratio: Ratio of V2/V1 Ratios (95% CI)	*p*	FDR-Adjusted *p*
Age		40	—	0.86 (0.72–1.02)	0.093	0.558
Sex	Female	7	1.32 (0.84–2.06)	0.95 (0.59–1.53)	0.839	0.876
	Male	33	1.25 (1.02–1.54)			
BMI		40	—	0.98 (0.81–1.17)	0.802	0.876
Creatinine		40	—	1.05 (0.87–1.25)	0.639	0.876
Preoperative AF	No	32	1.28 (1.04–1.58)	0.92 (0.58–1.44)	0.714	0.876
	Yes	8	1.18 (0.78–1.79)			
Previous MI	No	26	1.20 (0.95–1.50)	1.17 (0.80–1.70)	0.422	0.876
	Yes	14	1.40 (1.02–1.91)			
Surgical strategy	Off-pump	13	1.29 (0.93–1.79)	0.97 (0.66–1.43)	0.876	0.876
	On-pump	27	1.25 (1.00–1.57)			
LVEF		40	—	1.19 (1.00–1.42)	0.057	0.558
RAAS inhibitor	No	8	1.07 (0.71–1.62)	1.23 (0.78–1.92)	0.380	0.876
	Yes	32	1.31 (1.07–1.62)			
Loop diuretic	No	30	1.24 (1.00–1.53)	1.09 (0.72–1.65)	0.701	0.876
	Yes	10	1.34 (0.92–1.95)			
MRA	No	32	1.24 (1.01–1.53)	1.09 (0.69–1.71)	0.721	0.876
	Yes	8	1.35 (0.89–2.05)			
Amiodarone	No	26	1.36 (1.08–1.70)	0.81 (0.56–1.18)	0.289	0.876
	Yes	14	1.10 (0.81–1.51)			

Adjusted V2-to-V1 ratios with 95% confidence intervals are presented within each category of categorical predictors. The interaction ratio is defined as the change in the longitudinal V2-to-V1 ratio associated with a one-standard-deviation increase in a continuous predictor. For categorical predictors, the interaction ratio compares the V2-to-V1 ratio in the second category with that in the first category listed. The models were adjusted for age and sex, except when age or sex was itself the predictor, and included time, the candidate predictor, their interaction, and a subject-specific random intercept. Interaction *p* values were adjusted separately within each biomarker across the 12 included predictors using the Benjamini–Hochberg procedure. Abbreviations: AF, atrial fibrillation; BMI, body mass index; CI, confidence interval; FDR, false discovery rate; IL-18, interleukin-18; LVEF, left ventricular ejection fraction; MI, myocardial infarction; MRA, mineralocorticoid receptor antagonist; RAAS, renin–angiotensin–aldosterone system; V1, preoperative measurement; V2, three-month post-discharge follow-up measurement.

**Table 6:** Effect modifiers of longitudinal NT-proBNP changes.

Predictor	Group or Scale	N	Adjusted V2/V1 Ratio (95% CI)	Interaction Ratio: Ratio of V2/V1 Ratios (95% CI)	*p*	FDR-Adjusted *p*
Age		40	—	1.07 (0.84–1.37)	0.593	0.890
Sex	Female	7	1.39 (0.76–2.55)	1.07 (0.56–2.05)	0.828	0.937
	Male	33	1.50 (1.13–1.98)			
BMI		40	—	0.78 (0.62–0.99)	0.044	0.528
Creatinine		40	—	1.03 (0.80–1.32)	0.818	0.937
Preoperative AF	No	32	1.47 (1.11–1.95)	1.03 (0.56–1.89)	0.937	0.937
	Yes	8	1.51 (0.86–2.65)			
Previous MI	No	26	1.46 (1.06–1.99)	1.04 (0.62–1.74)	0.880	0.937
	Yes	14	1.52 (0.99–2.32)			
Surgical strategy	Off-pump	13	1.28 (0.83–1.99)	1.23 (0.73–2.07)	0.437	0.890
	On-pump	27	1.58 (1.16–2.15)			
LVEF		40	—	1.19 (0.94–1.52)	0.162	0.660
RAAS inhibitor	No	8	1.73 (0.98–3.03)	0.82 (0.45–1.51)	0.535	0.890
	Yes	32	1.42 (1.07–1.88)			
Loop diuretic	No	30	1.40 (1.05–1.88)	1.23 (0.70–2.16)	0.476	0.890
	Yes	10	1.72 (1.04–2.85)			
MRA	No	32	1.42 (1.07–1.88)	1.22 (0.66–2.24)	0.530	0.890
	Yes	8	1.73 (0.99–3.04)			
Amiodarone	No	26	1.30 (0.96–1.77)	1.44 (0.87–2.37)	0.165	0.660
	Yes	14	1.87 (1.23–2.84)			

Adjusted V2-to-V1 ratios with 95% confidence intervals are presented within each category of categorical predictors. The interaction ratio is defined as the change in the longitudinal V2-to-V1 ratio associated with a one-standard-deviation increase in a continuous predictor. For categorical predictors, the interaction ratio compares the V2-to-V1 ratio in the second category with that in the first category listed. The models were adjusted for age and sex, except when age or sex was itself the predictor, and included time, the candidate predictor, their interaction, and a subject-specific random intercept. Interaction *p* values were adjusted separately within each biomarker across the 12 included predictors using the Benjamini–Hochberg procedure. Abbreviations: AF, atrial fibrillation; BMI, body mass index; CI, confidence interval; FDR, false discovery rate; LVEF, left ventricular ejection fraction; MI, myocardial infarction; MRA, mineralocorticoid receptor antagonist; NT-proBNP, N-terminal pro-B-type natriuretic peptide; RAAS, renin–angiotensin–aldosterone system; V1, preoperative measurement; V2, three-month post-discharge follow-up measurement.

## Data Availability

The data are available upon reasonable request, as the dataset also contains unpublished data.

## Abbreviations

The following abbreviations are used in this manuscript:

CABG	coronary artery bypass grafting
NT-proBNP	N-terminal pro-B-type natriuretic peptide
hsCRP	high-sensitivity C-reactive protein
cTnI	cardiac troponin I
TNF-α	tumor necrosis factor alpha
IL-18BP	IL-18 binding protein
AF	atrial fibrillation
MI	myocardial infarction
RAAS	renin–angiotensin–aldosterone system
ARNI	angiotensin receptor–neprilysin inhibitor
LVESV	left ventricular end-systolic volume
LVEDV	left ventricular end-diastolic volume
LVEF	left ventricular ejection fraction
LAV	left atrial volume
TAPSE	tricuspid annular plane systolic excursion
